# An Efficient Genotyping Method for Genome-modified Animals and Human Cells Generated with CRISPR/Cas9 System

**DOI:** 10.1038/srep06420

**Published:** 2014-09-19

**Authors:** Xiaoxiao Zhu, Yajie Xu, Shanshan Yu, Lu Lu, Mingqin Ding, Jing Cheng, Guoxu Song, Xing Gao, Liangming Yao, Dongdong Fan, Shu Meng, Xuewen Zhang, Shengdi Hu, Yong Tian

**Affiliations:** 1 Laboratory of RNA Biology, Institute of Biophysics, Chinese Academy of Sciences, Beijing 100101, China; 2 University of Chinese Academy of Sciences, Beijing 100080, China; 3 These authors contributed equally to this work.

## Abstract

The rapid generation of various species and strains of laboratory animals using CRISPR/Cas9 technology has dramatically accelerated the interrogation of gene function *in vivo*. So far, the dominant approach for genotyping of genome-modified animals has been the T7E1 endonuclease cleavage assay. Here, we present a polyacrylamide gel electrophoresis-based (PAGE) method to genotype mice harboring different types of indel mutations. We developed 6 strains of genome-modified mice using CRISPR/Cas9 system, and utilized this approach to genotype mice from F0 to F2 generation, which included single and multiplexed genome-modified mice. We also determined the maximal detection sensitivity for detecting mosaic DNA using PAGE-based assay as 0.5%. We further applied PAGE-based genotyping approach to detect CRISPR/Cas9-mediated on- and off-target effect in human 293T and induced pluripotent stem cells (iPSCs). Thus, PAGE-based genotyping approach meets the rapidly increasing demand for genotyping of the fast-growing number of genome-modified animals and human cell lines created using CRISPR/Cas9 system or other nuclease systems such as TALEN or ZFN.

Laboratory animal models have significantly contributed to the interrogation of gene function in biological systems. In recent years, novel genome editing technologies have been applied to establish animal and human cell models at an increasing speed^1^,^2^. Compared with methods using Zinc Finger Nucleases (ZFN) or Transcription activator TAL-like Effector Nucleases (TALEN), a more recent technology called Clustered Regularly Interspaced Short Palindromic Repeats (CRISPR)/CRISPR-associated 9 (Cas9) has played an increasing role in generating single and multiplexed genome-modified animal models^3^,^4^,^5^,^6^,^7^,^8^. This method relies on injecting *Cas9* mRNA together with single guide RNA (sgRNA) into oocytes that were collected from super-ovulated animals. The Cas9 protein forms a complex with the sgRNA, upon which the cognate target is identified by the complex by recognition of a short trinucleotide NGG protospacer adjacent motif (PAM) sequence^9^,^10^. Subsequently, the catalytic activity of Cas9 causes scission of double-stranded DNA and generates double-strand breaks (DSBs) in target DNA. Cleaved DSBs will be repaired either by a non-homologous end joining (NHEJ) mechanism, which is error-prone and therefore generates indel mutations in the vicinity of DSBs, or by homologous recombination (HR), a process that can be exploited for more precise genomic modification^11^,^12^. So far, several reports have described the successful generation of genome-modified animal models from various species, including mouse^5^,^8^,^13^, rat^14^,^15^,^16^, zebrafish^17^,^18^, rabbit^19^, pig^20^ and monkey^21^.

As the number of genome-edited animal models and human cell models increases, genotyping is becoming a bottleneck, especially for high-throughput screening^22^,^23^. Techniques have been developed for the detection of indel mutations at the targeted loci, such as Surveyor assays (Cel1), T7 endonuclease 1 (T7E1) assays or High Resolution Melt Analysis (HRMA)^24^,^25^. Surveyor nuclease and T7E1 are mismatch-specific DNA endonucleases that are used for detecting indel mutations generated by genome engineering nuclease such as ZFN and TALEN^26^,^27^,^28^. The widely used T7E1 endonuclease targets and digests mismatched heteroduplex double-strand DNA, and as a result produces two or more smaller fragments in an enzymatic reaction. The digested DNA fragments can thus be resolved and visualized by agarose gel electrophoresis^8^. In addition, the re-hybridized fragments may also subjected to melting curve analysis (HRMA) that is part of the real-time thermocycler^25^. Fragments that contain mismatches melt at a lower temperature than perfect duplexes. To date, genotyping of genome-modified mouse and rat models generated using CRIPSR/Cas9 technology is primarily done using the T7E1 assay^29^,^30^. However, for genotyping of large-scale screening events like cell transfection or mouse embryo injections, the conventional T7E1 and HRMA assays are both time- and labor-consuming^23^,^31^. Therefore, it is highly desirable to design simple and efficient strategies for the genotyping of indel mutations, especially when required for high-throughput screening methods.

Here, we have applied a one-step polyacrylamide gel electrophoresis-based (PAGE) approach to successfully genotype 6 different strains of genome-modified animals and human induced pluripotent stem cells (iPSCs) harboring indel mutations caused by the CRISPR/Cas9 system. Compared to the traditional T7E1 assay, the PAGE-based approach proves to be a more efficient,time- and labor-saving strategy, without compromising sensitivity during the genotyping of CRISPR/Cas9-mediated indel mutations. Therefore, our one-step PAGE-based approach can replace the T7E1 assay as a routine laboratory protocol for genotyping laboratory animal models and human cell lines generated with the CRISPR/Cas9 system, but also with other nuclease systems, such as TALEN or ZFN.

## Results

### Schematic overview of heteroduplexed DNA detection using PAGE

**Figure 1:**
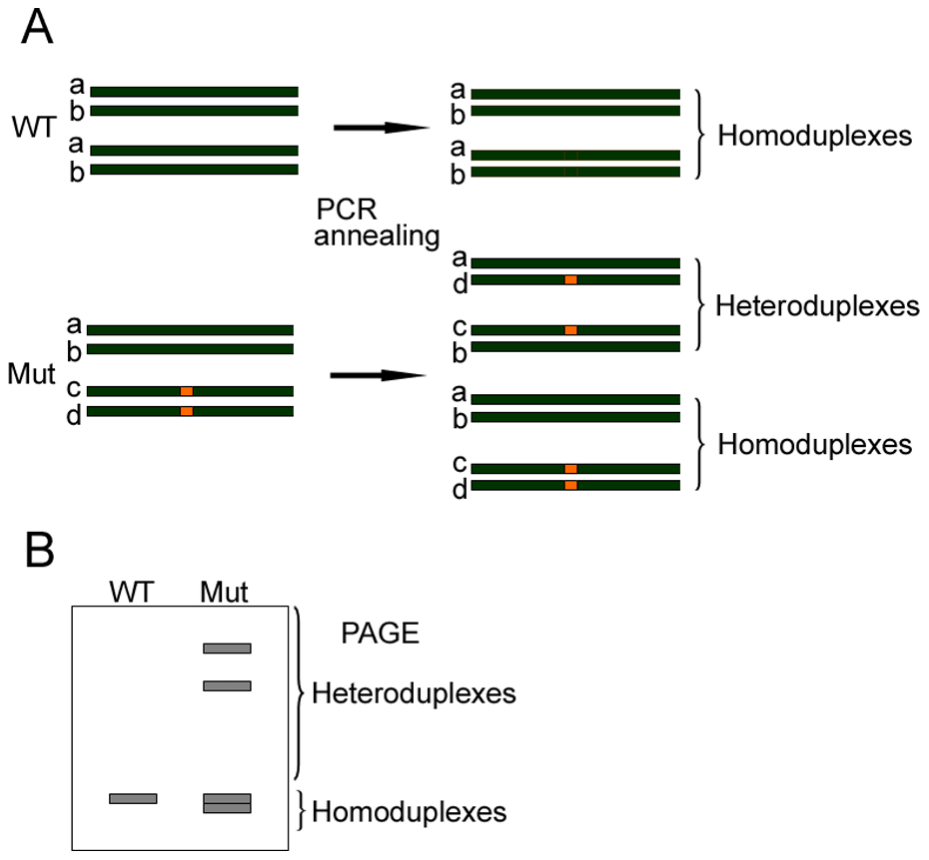
Schematic overview of PAGE-based genotyping protocol for identification of CRISPR/Cas9-mediated indel mutations. (A) Illustration of heteroduplex DNA formation during denaturation and annealing. Dark green bars represent four DNA strands (a–d) in cells harboring monoallelic mutations (orange box). After denaturation and annealing, two types of homoduplex DNA and two types of heteroduplex DNA were formed. (B) Identification of heteroduplex DNA fragments by 15% PAGE assay. Since heteroduplex DNA migrates slower due to formation of an open angle between matched and unmatched genomic regions, homoduplex DNA and heteroduplex DNA are easily identified based on their mobility rate.

PAGE-based assays have been used traditionally for the characterization of heteroduplex formation, especially for human viruses^32^,^33^. We first asked whether a PAGE-based approach can identify genome-modified mice carrying CRISPR/Cas9-mediated indel mutations with both high sensitivity and accuracy. In order to generate strains of genetically modified mice, we routinely isolated 100–250 zygotes collected from superovulated C57/BL6 mice and microinjected simultaneously *Cas9* mRNA and sgRNA prior to the transplantation of the zygotes into the oviducts of pseudo-pregnant ICR female mice. After DNA isolation from mouse tails in the F0 generation, we amplified the genomic regions spanning the sgRNA binding site using PCR. After brief denaturation and annealing, PCR fragments from genetically modified animals, which contain a mixture of indel mutations and wildtype alleles, formed heteroduplex DNA and homoduplex DNA (Figure 1A). Due to the existence of an open angle between matched and mismatched DNA strands caused by indel mutations, heteroduplex DNA generally migrated at a significantly slower pace than homoduplex DNA during native PAGE, thus making it a useful tool to screen founder colonies that harbor indel mutations (Figure 1B).

### Genotyping genome-modified mice generated by CRIPSR/Cas9 system by PAGE

**Figure 2:**
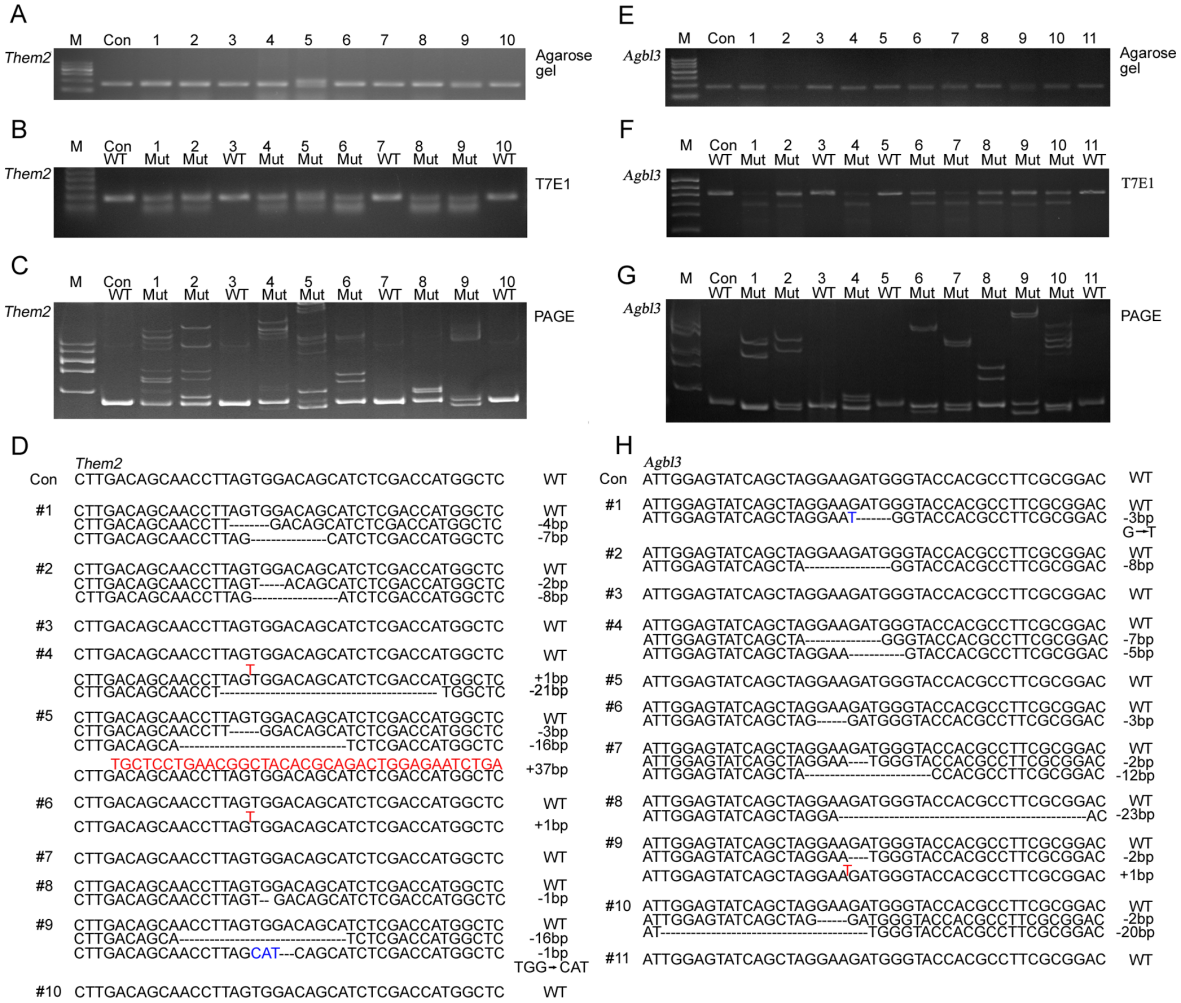
Detection of CRISPR/Cas9-mediated genome-modified mice targeting *Them2* and *Agbl3* loci by PAGE-based genotyping assay. (A) Identification of founder mice targeting the *Them2* locus from F0 generation by 2.5% agarose gel electrophoresis. (B) Identification of founder mice targeting the *Them2* locus by T7 endonuclease 1 (T7E1) cleavage assay. (C) 15% PAGE analysis detected heteroduplex DNA in mice #1, #2, #4, #5, #6, #8 and #9. Only homoduplex DNA was detected in mice #3, #7 and #10, similar to wildtype control. (D) Sequence analysis confirmed founder mice identified from Figure 2B harboring various types of indel mutations. (E) Identification of founder mice targeting the *Agbl3* locus from F0 generation using 2.5% agarose gel electrophoresis. (F) Identification of founder mice targeting the *Agbl3* locus using T7 endonuclease 1 (T7E1) cleavage assay. (G) Heteroduplex DNA was detected using 15% PAGE analysis in mice #1, #2, #4, #6, #7, #8, #9 and #10. Only homoduplex DNA was detected in mice #3, #5 and #11, as wildtype control. (H) Sequencing analysis confirmed founder mice identified from Figure 2G harboring one or two types of indel mutations. Base insertions are shown in red; base substitutions are shown in blue in Figure 2D and 2H. M, DNA marker; WT, wildtype; Con, control.

In order to validate the PAGE-based approach for use in our genotyping assay, we constructed DNA vectors expressing sgRNAs that target exon 2 of the *Them2* locus, which is driven by a T7 promoter (Supplementary Table S1). After purification of transcribed sgRNAs and *Cas9* mRNA *in vitro*, we microinjected *Cas9* mRNA and one sgRNA into the cytoplasm of zygotes as described in Table 1. Genomic DNA was extracted for genotyping from mouse tails at postnatal day 10. PCR products were amplified using primer pairs as listed in Supplementary Table S2. After brief denaturation and annealing, PCR products were subjected to 2.5% agarose gel electrophoresis or 15% PAGE. To compare the genotyping results obtained from the PAGE-based approach with that of the T7E1 cleavage assay, we further purified the same fraction of PCR products and subjected the DNA to T7E1 cleavage. On a 2.5% agarose gel, we detected a single band of similar size for all pups except that for mouse #5, confirming the successful amplification of PCR reactions (Figure 2A). PCR products from mouse #5 yielded one extra band in addition to the universal band generated from all other mice, indicating that a larger genomic fragment was modified. Using the T7E1 cleavage assay, efficient endonuclease activity was confirmed by identification of one extra digested band in 7 out of 10 pups. In mice #3, #7 and #10, we failed to detect any enzymatic digestion in comparison to other pups, suggesting wildtype alleles were maintained in these three mice (Figure 2B). To test the validity and accuracy of the PAGE-based approach, we performed this assay in parallel to the T7E1 cleavage assay. In wildtype control mice, PCR amplification yielded a 165-bp band on a 15% PAGE gel (Figure 2C). However, we detected multiplexed bands containing homoduplex DNA and heteroduplex DNA in 7 out of 10 pups analyzed. Pups #6 and #8 displayed a single pair of heteroduplex bands, indicating one dominant type of indel mutations exists in these two mice. Pups #3, #7 and #10 displayed band patterns identical to those found in wildtype controls, suggesting that no targeting was achieved in these three mice. Interestingly, samples from pups #1, #2 and #5 displayed multiplexed pairs of heteroduplex bands, suggesting that these three founders contain biallelic or tri-allelic mutations. To confirm our findings, we performed further TA cloning and sequencing (Figure 2D). Five clones from each mouse were randomly selected for sequencing. In wildtype control mice as well as in pups #3, #7 and #10, only sequences of wildtype alleles were identified. In addition, we failed to detect any Single Nucleotide Polymorphisms (SNP) near the sgRNA binding site. Targeted modifications within a size range of −16 bp and +37 bp occurred at the *Them2* locus in the remaining mice with variable efficiencies, thus confirming the results shown in Figure 2B and 2C.

In addition, we tested our PAGE-based genotyping approach by examining genome-modified mice by targeting sgRNA to exon 4 of the *Agbl3* locus. We analyzed a total of 11 animals from the F0 generation for identification of founder mice. On a 2.5% agarose gel, PCR reaction yielded a single band for all mice (Figure 2E). A subsequent T7E1 assay identified mice #3, #5 and #11 as wildtype, while all other mice exhibited bands cleaved by T7E1 (Figure 2F). In the 15% PAGE analysis, all mice except #3, #5 and #11 displayed various migration patterns of heteroduplex DNA (Figure 2G). Sequencing analysis confirmed that 9 out of 13 mice possessed different indel mutations (Figure 2H). However, mice #3, #5 and #11 yielded only wildtype homoduplex bands, which were confirmed by sequencing. Thus, our data suggest that genotyping based on PAGE achieves results consistent with the T7E1 cleavage assay.

### Genotyping multiplexed genome-modified mice generated by CRIPSR/Cas9 system with PAGE

**Figure 3:**
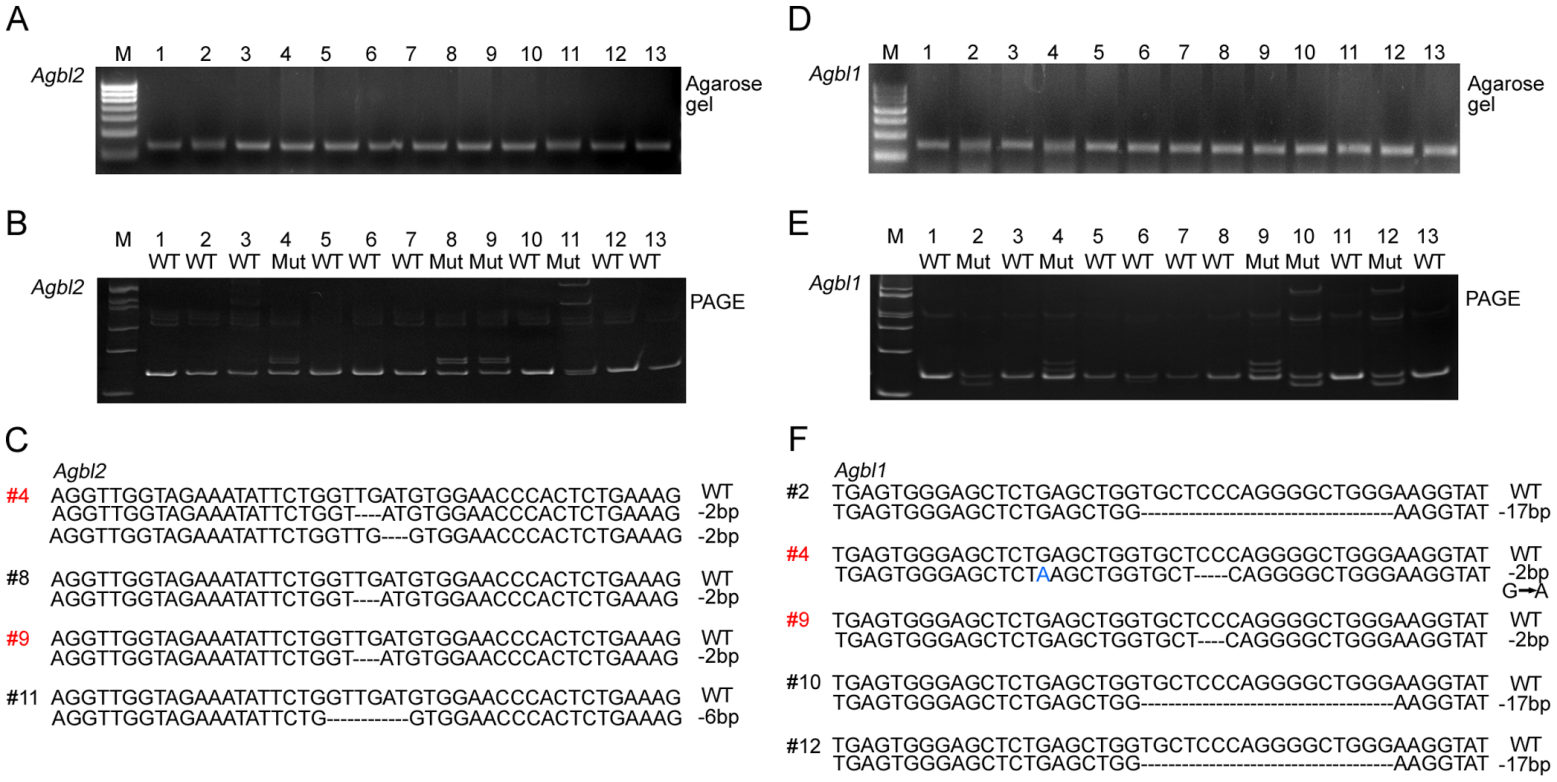
Identification of CRISPR/Cas9-mediated multiplexed genome-modified mice targeted to *Agbl1* and *Agbl2* loci by PAGE-based genotyping analysis. SgRNAs targeting *Agbl1* and *Agbl2* loci were mixed prior to microinjection into the cytoplasm of oocytes. Pups from F0 generation were genotyped for targeted modification in *Agbl1* and *Agbl2* loci. Results from agarose gel electrophoresis of PCR products are shown for the *Agbl2* (A) and *Agbl1* (D) loci. (B) Using the PAGE-based genotyping protocol, genomic indel mutations in the *Agbl2* locus were detected in mice # 4, #8, #9 and #11, while all others and wildtype controls exhibited only homoduplex DNA. (C) Sequence analysis confirmed the identification of indel mutations in mice #4, #8, #9 and #11 from Figure 3B. (E) PAGE analysis identified heteroduplex DNA from the *Agbl1* locus in mice #2, #4, #9, #10 and #12. (F) Sequencing analysis confirmed the identification of indel mutations in mice #2, #4, #9, #10 and #12 from Figure 3E. Note that mice #4 and #9 (shown in red) possessed indel mutations in both *Agbl2* and *Agbl1* loci. M, DNA marker; WT, wildtype; Mut, mutant.

We further examined multiplexed genome-modified mice from the F0 generation generated using simultaneous targeting of sgRNAs to both *Agbl2* and *Agbl1* loci*.* The relevant sgRNA sequences and injection conditions are listed in Table 1 and Supplementary Table S1. A single sgRNA was designed to target exon 7 of the *Agbl2* locus. PCR with genomic DNA of all 13 pups genotyped yielded one band of a 137-bp product, as shown by agarose gel assay (Figure 3A). Next, all PCR products were subjected to a 15% PAGE assay, and of those 13 pups, we identified mice #4, #8, #9 and #11 as containing a heteroduplex formation (Figure 3B). To confirm these results, we subjected all samples to TA cloning and sequencing (Figure 3C). Mice #4, #8 and #9 possessed an identical mutation, with a deletion of 2 base pairs, whereas mouse # 11 possessed a genomic deletion of 6 base pairs length. Within the *Agbl1* locus, a sgRNA that target exon 2 of the *Agbl1* gene was designed. As expected, all mice displayed similar band patterns when run on a 2.5% agarose gel (Figure 3D). On the 15% PAGE gel, 5 out of 13 mice displayed a homoduplex DNA band, identical to that observed for wildtype mice (Figure 3E). Furthermore, we observed a pair of heteroduplex DNA bands in mice #2, #4, #9, #10 and #12, which was confirmed by subsequent sequencing analysis (Figure 3F). Notably, both mouse #4 and #9 exhibited biallelic indel mutations in the two loci *Agbl2* and *Agbl1*. Together, these data suggest that a PAGE-based genotyping approach can indeed identify multiplexed indel mutations in mice.

### Genotyping F1 and F2 generations of genome-modified mice with PAGE

**Figure 4:**
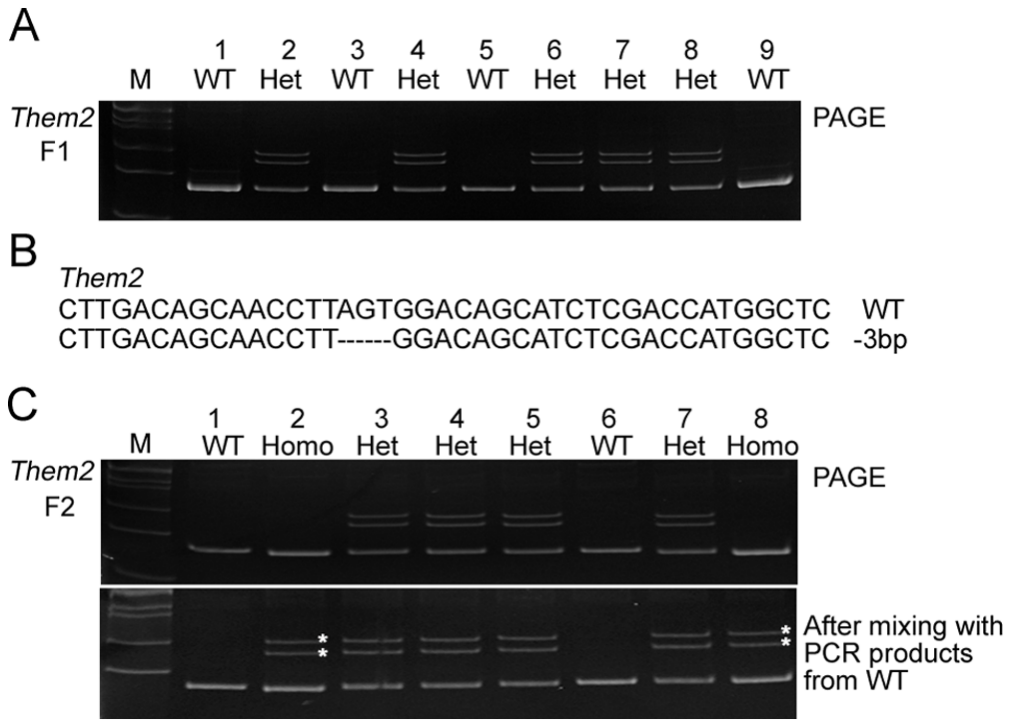
Identification of F2 generation homozygous *Them2* mutant mice via PAGE-based genotyping protocol. Mouse #5 (Figure 2D) were bred with wildtype C57/BL6 mice to obtain F1 mice. We further intercrossed mice from F1 to obtain homozygous F2 mutant mice. (A) Identification of F1 offsprings using PAGE-based genotyping approach. Five out of nine F1 offsprings were identified by PAGE analysis as carrying one type of indel mutations in the *Them2* locus. (B) Sequencing results from F1 offsprings revealed mice harboring a 3-bp deletion. (C) Identification of F2 offsprings using PAGE-based genotyping approach. F2 mice #3, #4, #5 and #7 harboring heterozygous indel mutations in their *Them2* locus were identified by PAGE assay (upper panel). When mixing PCR products from control mice with PCR products from F2 mice, mice #2 and #8 exhibited migration patterns from heteroduplex DNA (marked by asterisks), indicating homozygous indel mutation (bottom panel). M, DNA marker; WT, wildtype; Homo, homozygote; Het, heterozygote.

Given the genotyping results described above, we expected that a PAGE-based protocol can also be utilized for genotyping of genome-modified mice from F1 and F2 generations. To test this, we produced F1 generation offsprings by crossing founder #5 (Figure 2A) with wildtype C57/BL6 mice (Figure 4A). Genotyping of that generation by 15% PAGE analysis revealed that 5 out of 8 mice harbored the desired indel mutations. For verification, all PCR products from mice harboring the desired indel mutations were subjected to sequencing (Figure 4B). In addition, we bred mice carrying the 3-bp deletion mutation from the F1 generation, and screened F2 generation offsprings for identification of homozygous monoallelic animals. We hypothesized that it would not be possible to identify homozygous indel mutations, unless PCR products from wildtype alleles were mixed with mutant alleles. We first performed genotyping analysis by loading PCR samples directly onto 15% PAGE without prior mixing. As shown in Figure 4C, we only detected heterozygote mice rather than homozygous monoallelic mice. However, after mixing of the PCR products of mutants with controls prior to denaturation and annealing, it was possible to identify homozygous mice and wildtype mice (Figure 4C). Taken together, these results suggest that our PAGE-based protocol can be applied to genotyping of any generation of mice carrying indel mutations.

### Detection of various heteroduplexed DNA with PAGE

**Figure 5:**
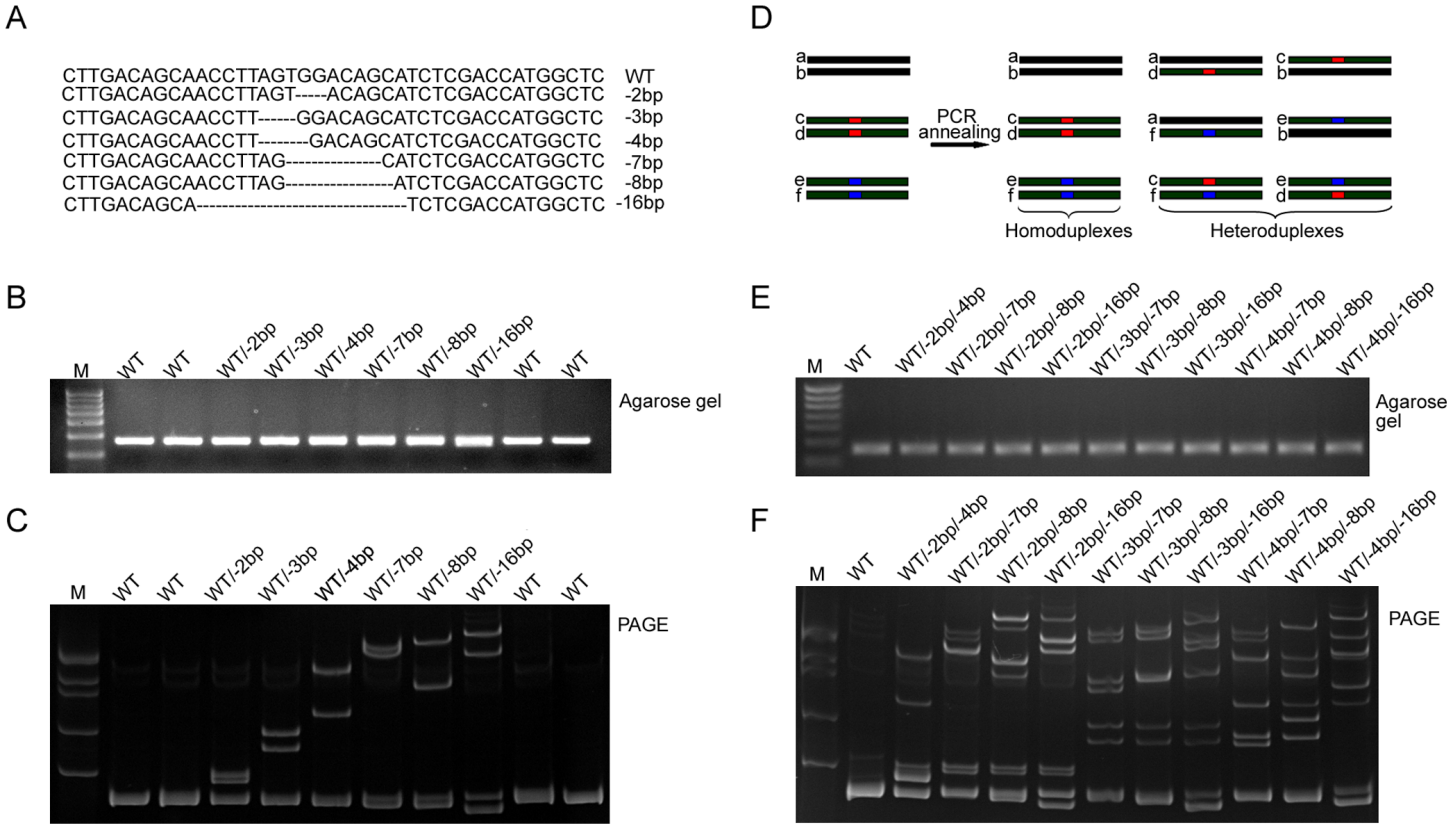
Illustration of heteroduplex DNA formation from plasmids harboring various indel mutations by PAGE analysis. (A) Sequence analysis identified DNA plasmids from *Them2* locus harboring -2 bp, -3 bp, -4 bp, -6 bp, -7 bp, -8 bp and -16 bp genomic deletion. (B-C) Agarose gel (B) and PAGE-based approach (C) for detection of various heteroduplex DNA. Agarose gel electrophoresis failed to detect indel mutations when mixing plasmids from wildtype allele with plasmids harboring various indel mutations; PAGE analysis revealed heteroduplex DNA formation as expected. (D) Schematic overview of heteroduplex DNA formation in case of samples harboring biallelic indel mutations. Red and blue boxes represent two type of indel mutations. After denaturation and annealing of PCR products, three types of homoduplex DNA and six types of heteroduplex DNA were formed. (E–F) Agarose gel (E) and PAGE-based approach (F) for detection of various combinations of heteroduplex DNA. PAGE analysis revealed heteroduplex DNA formation when mixing plasmids harboring wildtype allele with plasmids harboring two types of indel mutations, whereas agarose gel electrophoresis failed to detect any indel mutation.

To better understand the process of heteroduplex formation and its migration pattern for PAGE analysis, we utilized an array of plasmids carrying indel mutations of the *Them2* gene including deletions of 2 bp, 3 bp, 4 bp, 7 bp, 8 bp and 16 bp (Figure 5A). As shown in Figure 5B, all plasmids with various indel mutations yielded PCR products of similar size. After mixing PCR products from mutant and wildtype plasmids followed by denaturation and annealing, all indel mutations analyzed were able to produce distinct migration products on a 15% PAGE gel (Figure 5C). Noticeably, we were able to detect two types of homoduplex DNA in the lanes containing wildtype and -7 bp,-8 bp and -16 bp genomic deletion fragments individually. Since F0 mice may harbor biallelic or more complicated mosaic mutations, we hypothesized that each type of indel mutation should maintain their specific heteroduplex migration pattern on the PAGE gel (Figure 5D). We combined PCR products of any two types of biallelic mutations from the *Them2* locus, and observed similar sizes of PCR products among all samples when loaded onto an agarose gel (Figure 5E). Compared with their corresponding lanes in Figure 5C, different combinations of PCR products carrying biallelic mutations exhibited specific heteroduplex patterns on the 15% PAGE gel (Figure 5C and 5F). Thus, our data suggest that PAGE analysis can be used to assess the number and even types of indel mutations simply based on their heteroduplex mobility pattern.

### Sensitivity assessment of PAGE-based genotyping approach

**Figure 6:**
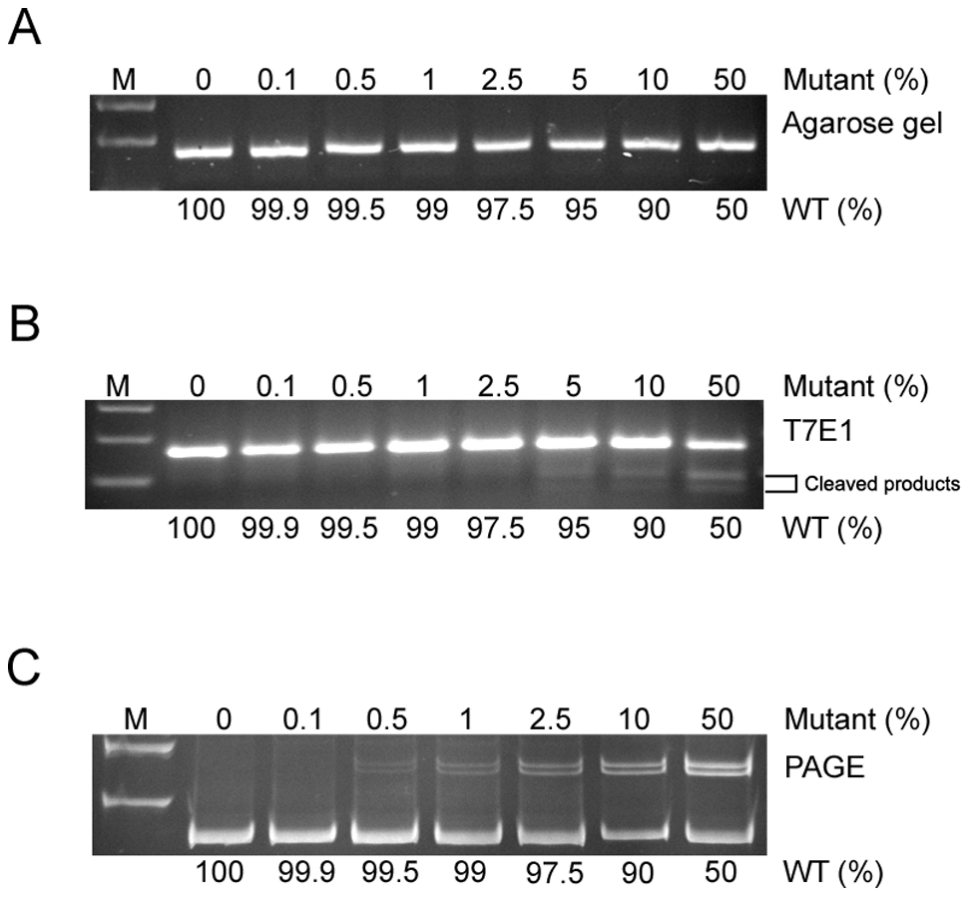
Sensitivity analysis for PAGE-based genotyping approach. (A) A total of 25 ng PCR template per reaction containing various ratios of wildtype and mutant clone DNA from the *Agbl3* locus were tested in a 50 μl volume. Equal volumes of PCR products were loaded in each well of 2.5% agarose gel. (B) PCR products were analyzed using the T7E1 assay. (C) PCR products were further analyzed using PAGE-based approach. The minimal detection percentage for mosaic DNA templates in this assay was similar between T7E1 and PAGE-based approach, as shown in Figure 6B and 6C.

To assess the sensitivity of PAGE-based genotyping approach, we performed PCR reactions with a total of 25 ng DNA template containing various ratios of wildtype and mutant clone DNA from the *Agbl3* locus, mixed in a 50 μl PCR reaction volume (Figure 6). Primer pairs for amplifying *Agbl3* locus were listed in Supplementary Table S2. PCR products of wildtype 166 bp and mutant 163 bp were used as loading controls on the agarose gel, and equal amounts of PCR products of various ratios were loaded into each well (Figure 6A). PCR products were further subjected to T7E1 or PAGE analysis after denaturation and annealing. For the T7E1 assay, the minimal detection percentage for mosaic DNA templates was between 0.5 to 5% (Figure 6B). For the PAGE-based assay, the maximal detection sensitivity was determined as 0.5% (Figure 6C), which is similar to that of T7E1 assay according to a previous study^34^. Because the T7E1 assay allows quantification of indel frequencies by measuring the intensities of cleaved DNA bands, we further quantified heteroduplexed bands from Figure 6C and calculated the correlation coefficient (Figure S3). Together, our data showed that overall sensitivity for detecting mosaic DNA harboring indel mutations using a PAGE-based approach was similar to that of T7E1 assay.

### Screening CRISPR/Cas9-mediated on- and off-target mutations in human 293T and induced pluripotent stem cells with PAGE

**Figure 7:**
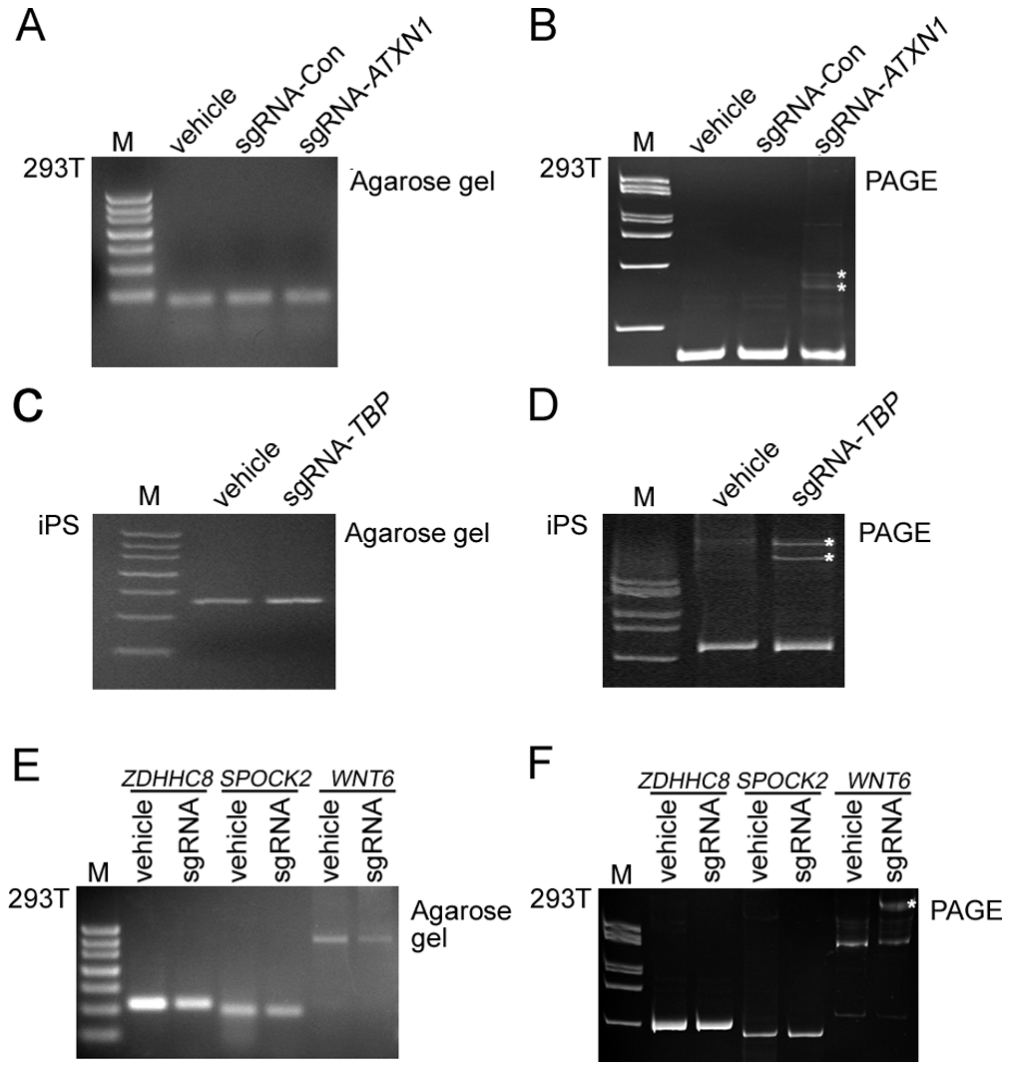
Identification of on- and off-target indel mutations from human 293T and iPSCs using PAGE-based genotyping protocol. (A) On-target screening in 293T cells using agarose gel electrophoresis. 293T cells were transfected using Cas9-GFP plasmids with either vehicle (no sgRNA), sgRNA-con (control sgRNA targeting a third locus (data not shown) or sgRNA-*ATXN1* (sgRNA targeting the *ATXN1* locus). Two days post transfections, cells expressing eGFP were sorted by flow cytometry prior to DNA extraction. (B) On-target screening in 293T cells using the PAGE-based approach. PAGE-based genotyping protocol detected heteroduplex DNA formation in cells transfected with sgRNA-*ATXN1*, but not in controls. (C) On-target screening in iPSCs using agarose gel electrophoresis. Human iPSCs were electroporated with either vehicle (no sgRNA) or sgRNA-*TBP* (sgRNA targeting the *TBP* locus). (D) On-target screening in human induced pluripotent stem cells using PAGE-based approach. (E) Off-target screening in 293T cells using agarose gel electrophoresis. 293T cells were transfected using Cas9-GFP plasmids with either vehicle (no sgRNA), sgRNA-con (control sgRNA targeting a third locus or sgRNA-*ATXN2* (sgRNA targeting the *ATXN2* locus) for screening off-target loci. Three off-target loci including *ZDHHC8*, *SPOCK2* and *WNT6* were identified using CRISPR Design Tool (http://crispr.mit.edu). (F) Off-target screening in 293T cells using PAGE-based analysis. Note that off-target effect was only detected in the *WNT6* locus, not in controls or other loci tested. Heteroduplex DNA bands were marked by asterisks in (B), (D) and (F).

To define whether our PAGE-based genotyping approach can be employed in human cell lines harboring indel mutations, plasmids expressing Cas9-GFP together with sgRNAs targeting either *ATXN1* or *ATXN2* locus were transfected into human 293T cell lines. Vehicle only (i.e. lacking sgRNA) and sgRNA targeting a third locus were used as controls in parallel. Two days post transfection, cells expressing GFP were sorted by flow cytometry, and DNA was extracted for genotyping. Oligos for sgRNA synthesis and genotyping were designed as listed in Supplementary Table S1 and S3. As expected, PCR products of similar size were detected for all samples. Using PAGE analysis, we were able to identify one pair of heteroduplex bands in the sgRNA-*ATXN1* lane (marked by asterisks), while no similar band was observed in the controls (Figure 7B). We next electroporated either sgRNA targeting the *TBP* locus (sgRNA-*TBP*) or vehicle control (Vehicle), together with a Cas9-GFP plasmid into human iPSCs. Ten days post transfection, iPSCs were examined for on-target screening. Single bands of similar sizes were detected on agarose gel (Figure 7C). PAGE-based analysis detected clear heteroduplex DNA in the sgRNA-*TBP* group (marked by asterisks), not, however, in the vehicle-only group (Figure 7D). Since off-target effects are of major concern when employing the CRISPR/Cas9 system, especially for use in human stem cell research, we wondered if off-target screening could be performed using our PAGE-based assay. To address this issue, we used a novel sgRNA targeting the human *ATXN2* locus with a predicted strong off-target effect. All potential off-target sites were identified using the CRISPR Design Tool (http://crispr.mit.edu) and BLAST algorithm, as described previously^35^. We identified three off-target sites, namely *ADHHC8*, *SPOCK2* and *WNT6*, all of which possessed 1 to 3 mismatches to the original *ATXN2* sgRNA sequences (Supplementary Table S3). PCR products from all three genes revealed a single band of similar size when loaded onto an agarose gel (Figure 7E, Supplementary Table S2). Using PAGE analysis, we identified heteroduplex DNA bands in the *WNT6* locus (marked with an asterisk), which was absent in the controls (Figure 7F). We also examined off-target loci in human iPSCs using our PAGE-based approach. No off-target effects were detected within any of the predicted loci in iPSCs transfected with sgRNA-*TBP* (Supplementary Figure S2 and Table S4). All off-target screening results were confirmed by sequencing (data not shown). Taken together, our data suggest that PAGE-based genotyping is an efficient method for analyzing indel mutations, as well as for screening off-target effects in human cells.

## Discussion

We present an efficient one-step method for the genotyping of indel mutations created using the CRIPSR/Cas9 system, in a variety of mouse strains and human cell lines. Our data show that this PAGE-based approach can detect different types of indel mutations, with both high sensitivity as well as efficiency. First, the one-step PAGE-based genotyping approach does not require an enzymatic reaction, which can produce false negative results due to incomplete digestion of mismatched DNA fragments. Instead, the PCR products are directly subjected to PAGE-based electrophoresis for mutant allele detection. Second, our PAGE-based approach can detect mosaic indel mutations with similar sensitivity to that of a conventional T7E1 assay. Based on our findings, the PAGE-based assay can detect 0.5% to 5% mutant DNA composition, which is in agreement with a previous report^36^. Third, since different heteroduplex complexes of indel mutations display specific motility pattern when run on a PAGE gel, the number of different types of mutations can be directly assessed from evaluating the stained gel, which provides additional information not available with other methods^25^. Fourth, manipulating genome-modified human iPS cells by using the CRISPR/Cas9 system requires analysis of both on- and off-target effects. The genome-wide binding of Cas9 protein raises considerable concern over the off-target issue and has been a significant obstacle for applying genome-editing tools towards regenerative medicine^37^,^38^. Off-target sites can tolerate up to five mismatches to the sgRNA sequence and many were mutagenized with frequencies comparable to the intended on-target site due to the genome-wide binding^38^,^39^. Our data suggest that the PAGE-based genotyping approach is efficient for screening on- and off-target effects in human iPS cells, and importantly, can do so in a high-throughput manner. Fifth, unlike the RGEN-RFLP approach that allows more precise quantification with a reported R^2^ = 0.99 at least in one study^36^, both T7E1 and PAGE methods failed to achieve R^2^ close to 0.99 (Figure S3). This indicates that results of the PAGE and T7E1 assay correlated poorly with mutation frequencies. Thus, for quantification purposes, RGEN-RFLP is the method of choice and PAGE-based assay is not suitable for quantification of indel mutations. Finally, although RGEN-RFLP method can be used to genotype mutations, it does involve multiple steps, such as purification of Cas9 protein, transcription of sgRNA, as well as *in vitro* enzymatic incubation steps^36^. Evidently, PAGE-based approach provides a cost- and labor-saving strategy suitable for low-budget laboratories for relevant genotyping assay. However, if the target genomic DNA fragment contains single nucleotide polymorphisms (SNPs) or allelic mutations, both PAGE-based and T7E1 approach may give rise to false positive results. Under such cases, combining our PAGE method with a RGEN-RPLP approach can overcome this limitation and faithfully detect indel mutations caused by CRISPR/Cas9 system^36^.

In summary, the use of a one-step PAGE-based approach for genotyping of CRISPR/Cas9-mediated indel mutations proves to be a simple and efficient strategy with high sensitivity. This strategy can be applied to any animal model or human cells to detect on- or off-target indel mutations. It should be the ideal method of choice to meet the rapidly increasing demand for genotyping of a fast-growing number of genome-modified animals and human cell lines, and thus can be used as routine laboratory protocol for screening indel mutations generated by CRISPR/Cas9-system, as well as other nucleases.

## Methods

### Animal models

Mice colonies were maintained in standard cages in a SPF animal facility on a daily 12-hour light/dark cycle. All animal protocols were approved by the Institutional Animal Care and Use Committee (IACUC) at the Institute of Biophysics, Chinese Academy of Sciences.

### DNA vector preparation

Cas9 expression vector (pST1374-N-NLS-flag-linker-Cas9) for *in vitro* transcription and Cas9-GFP vector for human 293T cell transfection were obtained from Addgene (Addgene no. 44758 and 44719). For sgRNA expression in human cells and mouse gene targeting, DNA constructs were obtained from Addgene (Addgene no. 51132 and 51133). Synthesized oligos for sgRNA expression were denatured at 95°C for 5 minutes and annealed at room temperature, before being cloned between two BsaI sites of a linearized PUC57-sgRNA expression vector containing T7 or U6 promoter. The oligo sequences used for sgRNA synthesis are listed in Supplementary Table S1.

### *In vitro* transcription

DNA vector expressing *Cas9* mRNA was linearized by Agel or XmaI enzyme. *Cas9* mRNA was obtained using mMESSAGE mMACHINE T7 kit (Life Technologies, AM1344). Vectors for sgRNA expression were linearized by DraIII and *in vitro* transcribed using MEGAshortscript T7 kit (Life Technologies, AM1354). The transcribed sgRNA was further purified by pheno-chloroform and precipitated in cold ethanol followed by elution in RNase-free water. *Cas9* mRNA was also purified with RNeasy Mini kit (Qiagen, 74104) for embryo microinjection.

### Microinjection

Superovulated female C57/BL6 mice were mated to male C57/BL6 mice, and fertilized eggs were collected from the oviducts. *Cas9* mRNA (150 ng/μl) and transcribed sgRNA (100 ng/μl) were mixed and microinjected into the cytoplasm of fertilized eggs with well-recognized pronuclei in M2 medium (Sigma). Approximately 100–250 zygotes were injected with each corresponding sgRNA and subsequently transferred to the uterus of pseudo-pregnant ICR females, from which viable founder mice were obtained. Detailed information are summarized in Table 1.

### Mouse genomic DNA preparation

Mouse tail biopsies were digested overnight using 0.5 mg/ml protein kinase K (Roche, 03508838) in lysis buffer (50 mmol/L Tris-Cl, 100 mmol/L EDTA, 100 mmol/L NaCl, and 1% SDS). On the following day, a 5 M NaCl solution was added before pelleting tail debris at 17,000 g for 10 minutes. Then the supernatant containing DNA was precipitated in cold ethanol and resuspended in ddH_2_0. PCR was performed using primer pairs listed in Supplementary Table S2. The typical 50 μL PCR reaction mix contains 1 U Taq DNA Polymerase, 0.4 μM forward and reverse primer pairs, 1.5 mM MgCl_2_, 200 μM dNTP mix and genomic DNA template (<1 μg). The standard PCR condition was as follows: 94°C for 5 min; 94°C for 30 s, 58°C for 30 s, 72°C 30 s for 35 cycles; 72°C for 5 min followed with denaturation for 5 minutes at 95°C. PCR products were removed from the thermocycler and maintained at room temperature for at least 5 minutes allowing for annealing, before loading onto 15% polyacrylamide gel. For control purposes, a fraction of PCR products were resolved with ethidium bromide-stained 2.5% agarose gel.

### PAGE analysis

Direct-load PCR Marker (GenStar, M1201) or DNA Marker 1 (Biomed, DM0601) was used for each gel. Washed plates with 1.5 mm spacer were assembled for casting the acrylamide gel. The annealed PCR products were resolved by electrophoresis in non-denaturing polyacrylamide gels containing 15% acrylamide-bisacrylamide (29:1, w/w), 1X Tris-borate-EDTA (TBE), ammonium persulfate, and TEMED. After 2 hours of electrophoresis at 150 V, 33-37 mA, polyacrylamide gel was immersed in 0.5% ethidium bromide solution for 10 minutes before visualization using Geldoc XR+ Imaging System (Biorad). Purified PCR samples with positive heteroduplex bands were subjected to TA cloning and sequencing for confirmation.

### T7E1 cleavage assay

PCR amplicons from targeted genomic region were purified with Qiaquick PCR Purification Kit (Qiagen, 28106). For T7E1 cleavage assay, purified PCR products were denatured and annealed in NEBuffer 2 (NEB) using a thermocycler. Hybridized PCR products were digested with T7 endonuclease 1 (NEB, M0302L) for 30 minutes at 37°C and subjected to 2.5% agarose gel electrophoresis. All PCR primer sequences are listed in Supplementary Table S2.

### SgRNA design and identification of off-target sites

For mouse targeting, sgRNA target sites were selected with the sequence 5′-N_(19)_GG or 5′-N_(21)_GG (Supplementary Table S1). The artificial sequence GG were added to the 5′ end during oligo synthesis to provide essential BsaI sites for ligation into sgRNA expression vectors. For validation of sgRNA site and putative off-target sites identification, sgRNA plus PAM sequences were searched using BLAST algorithm (http://www.ensembl.org/Multi/blastview) and CRISPR Design Tool (http://crispr.mit.edu) against mouse genome assembly mm9 and human genome assembly hhg9. All possible off-target sites were screened by ungapped alignment, allowing for up to four mismatches in the target sgRNA sequence (Supplementary Table S3 and S4). Identified off-target loci were amplified by primers listed in Supplementary Table S2. PCR products were purified with a Qiaquick PCR Purification Kit (Qiagen, 28106) and subjected to 15% PAGE analysis or TA cloning for sequencing. Only indel mutations around the third bases upstream PAM sequence were considered as NHEJ-induced indel mutations.

### Human cell culture and gene targeting

Human embryonic kidney (HEK) cell line 293T cells were cultured in Dulbecco's modified Eagle's Medium (DMEM)-high glucose (Hyclone, SH30022.01) supplemented with 10% FBS. Approximately 5 × 10^6^ cells were co-transfected with 8 μg of Cas9-GFP vector, 2 μg of sgRNA expression vector using Lipofectamine 2000 according to the manufacturer's instructions (Invitrogen, 11668-019). Cells were sorted by flow cytometry (BD Biosciences, BD FACS AriaII) 48 hours post transfection and collected for genomic DNA extraction.

Human iPSCs were cultured on mouse embryonic fibroblast feeder cells in Knockout DMEM/F12 supplemented with 20% KOSR, 0.1 mM nonessential amino acids, 2 mM Glutamax, 1% penicillin/streptomycin, 55 μM β-mercaptoethanol and 7 ng/ml bFGF (R&D, 233-FB-01m). Media was changed daily, and cells were passaged every 6 to 7 days.

For electroporation, approximately 3 × 10^6^ human iPSCs were digested to single cells using accutase (Gibco, 17104-019), washed once with PBS and resuspended in 500 μl of PBS. 9 μg of Cas9-GFP vectors and 3 μg of sgRNA expression vectors were mixed with the cells before electroporation in a 4-mm cuvette. Electroporation parameters were set to 250 V, 500 μF and infinite resistance. Cells were then plated onto feeders with 10 μM ROCK inhibitor Y-27632 (Sigma, Y0503). Two days post transfection, cells expressing GFP were sorted with flow cytometry (BD Biosciences, BD FACS AriaII) and cultured on matrigel-coated 6-well plates in mTeSR medium (Stemcell Technologies, 05850) with 10 μM Y-27632 for one week before isolation for genomic DNA extraction.

## Author Contributions

Y.T. and S.H. designed the study; X.Z., Y.X., L.L., X.G., S.Y. and M.D. performed most of the experiments; J.C. performed microinjection; G.S., D.F. and X.Z. performed human iPS experiments; L.Y. and S.M. helped with mice maintenance and breeding; Y.T. and S.H. wrote the manuscript with the help from all authors; Y.T. and S.H. supervised the research.

## Supplementary Material

Supplementary Information Supplementary Materials

## Figures and Tables

**Table 1:** Summary of mouse embryo microinjection of Cas9 mRNA with different sgRNAs and number of mutant pups identified using the PAGE-based genotyping assay

Gene name	Cas9 (ng/μl)	sgRNA (ng/μl)	Injected zygotes	Newborns	Mutant pups identified with PAGE assay
*Agbl1*	150	100	116	13	5
*Agbl2*	150	100	116	13	4
*Agbl3*	150	100	121	14	9
*Agbl5*	150	100	64	5	3
*Nmi*	150	100	180	32	26
*Them2*	150	100	235	19	16
