# Supplementary material for: An Efficient Genotyping Method for Genome-modified Animals and Human Cells Generated with CRISPR/Cas9 System
Source: Sci Rep. 2014 Sep 19;4:6420. doi: 10.1038/srep06420 (PMC4168274; doi:10.1038/srep06420)
Supplement: Supplementary Information — Supplementary Materials [file srep06420-s1.pdf]

Supplementary Materials

An Efficient Genotyping Method for Genome-modified Animals and Human Cells Generated with CRISPR/Cas9 System

Xiaoxiao Zhu[1,2]*, Yajie Xu[1]*, Shanshan Yu[1]*, Lu Lu[1,2], Mingqin Ding[1,2], Jing Cheng[1], Guoxu Song[1,2], Xing Gao[1], Liangming Yao[1], Dongdong Fan[1,2], Shu Meng[1], Xuewen Zhang[1], Shengdi Hu[1‡] and Yong Tian[1‡]

[1]*Laboratory of RNA Biology, Institute of Biophysics, Chinese Academy of Sciences, Beijing, China 100101*

[2]*University of Chinese Academy of Sciences, Beijing 100080, China*

‡To whom correspondence should be addressed: Yong Tian & Shengdi Hu, Laboratory of RNA Biology, Institute of Biophysics, Chinese Academy of Sciences, 15 Datun Road, Beijing, China, 100101; phone: 86-10-64888579; E-mail: ytian@ibp.ac.cn (Y.T.); shengdihu@moon.ibp.ac.cn (S.H.).

*Authors contributing equally to this work

Running title: **Genotyping with CRISPR/Cas9**

Target Name	Direction	Sequence (5' to 3')
Agbl1	F	TAGGAGCTCTGAGCTGGTGCTCCC
	R	AAACGGGAGCACCAGCTCAGAGCT
Agbl2	F	TAGGTAGAAATATTCTGGTTGATG
	R	AAACCATCAACCAGAATATTTCTA
Agbl3	F	TAGGAGTATCAGCTAGGAAGAT
	R	AAACATCTTCCTAGCTGATACT
Agbl5	F	TAGGTTCTACTTCAGTGTCCGGGG
	R	AAACCCCCGGACACTGAAGTAGAA
Nmi	F	TAGGAAAACAAAGAACTAGACG
	R	AAACCGTCTAGTTCTTTGTTTT
Them2	F	TAGGTCGAGATGCTGTCCACTA
	R	AAACTAGTGGACAGCATCTCGA
ATXN1	F	TAGGGGGCAGTCTGAGCCAGACGC
	R	AAACGCGTCTGGCTCAGACTGCCC
ATXN2	F	TAGGGCAGCAGCAGCCGCCGCCCG
	R	AAACCGGGCGGCGGCTGCTGCTGC
TBP	F	TAGGGAGCAGGAACATAACTCAAA
	R	AAACTTTGAGTTATGTTCCTGCTC

Forward (F); Reverse (R).

Table S1. Oligonucleotides for generating mouse and human sgRNA expressing vectors.

Name	Direction	Sequence (5' to 3')	Product Size
Agbl1	F	ACAGTGTTACCCTGCGAGTC	140 bp
	R	TGCAAACACTCGTCTTAGAGC	
Agbl2	F	TGTGCTCTGAAAATCATTTCTTACT	137 bp
	R	TACCTTGCTCCTCTCCCACA	
Agbl3	F	TCAGCTGATTCTATTGGTGACCC	166bp
	R	TGACCTCACAGTGGTATGGC	
Agbl5	F	CATCCTCTCCCTGGCCCT	159 bp
	R	CCATGCCCTGGGAATACAGT	
Nmi	F	CTTAGGGGAGGGAGATTGGC	180 bp
	R	TGGAATTCTCTGGCATCCGA	
Them2	F	TGAAGGTGGAAGAGCAGCAT	165 bp
	R	CGGGATGGCCTCTGGATAC	
TBP	F	CCACAGCTCTTCCACTCACA	249 bp
	R	TAGTGCCACTCCCTCCCTTA	
KCNE1	F	CTGGAGCTCAACCAGGAGAA	209 bp
	R	AGCAGAGGGTGCCTAACTGA	
SEC16A	F	GCACGGGAATTGTCTTGAAT	230 bp
	R	CTGTCCAATCCAAGCTGTCA	
ATXN1	F	CCAGCTGGAGGCCTATTC	73 bp
	R	CTCAGCCTTGTGTCCCGG	
SPOCK2	F	GCAGGAGACACAGGCGCT	189 bp
	R	AGGAACGTCGAAGTGGAGTT	
WNT6	F	GGGTGGGAGGAAGGACATTA	456 bp
	R	GTCCTCCATGAAATTGCCGG	
ZDHHC8	F	CGCCCGGTCCACCTTAAG	244 bp
	R	TGTACTTGGCGGGTTTGAGG	

Forward (F); Reverse (R).

Table S2. Summary of primer sequences and PCR product size for identification of indel mutations in different strains of genome-modified mice and human cells.

Sequence (5’-3’)	Mismatch Number	Gene Name
**GCAGCAGCAGCCGCCGCCCG CGG**	0	*ATXN2*
GCAGCAGCCGCCGCCGCCCG TGG	1	*ZDHHC8*
GCAGCAGCAGCAGCAGCCCG AGG	2	*WNT6*
GCGGCAGCACCAGCCGCCCG CAG	3	*SPOCK2*

Note: Letters in red color represent mismatch bases in comparison to sgRNAs. Letters in bold represent sgRNAs and PAM sequences used for CRISPR/Cas9-mediated genetic editing in the *ATXN2* locus. PAM sequence refers to 5’-NGG or 5’-NAG.

Table S3. Putative on- and off-target cleavage sites for sgRNA -*ATXN2.*

Sequence (5’-3’)	Mismatch Number	Gene Name
**GAGCAGGAACATAACTCAAA GGG**	0	*TBP*
TAGAATCAACATAACTCAAA AGG	4	*SEC16A*
GACCAGAAGGATAACTCAAA AAG	4	*KCNE1*

Note: Letters in red color represent mismatch bases in comparison to sgRNAs. Letters in bold represent sgRNAs and PAM sequences used for CRISPR/Cas9-mediated genetic editing in the *TBP* locus. PAM sequence refers to 5’-NGG or 5’-NAG.

Table S4. Putative on- and off-target cleavage sites for sgRNA-*TBP*.

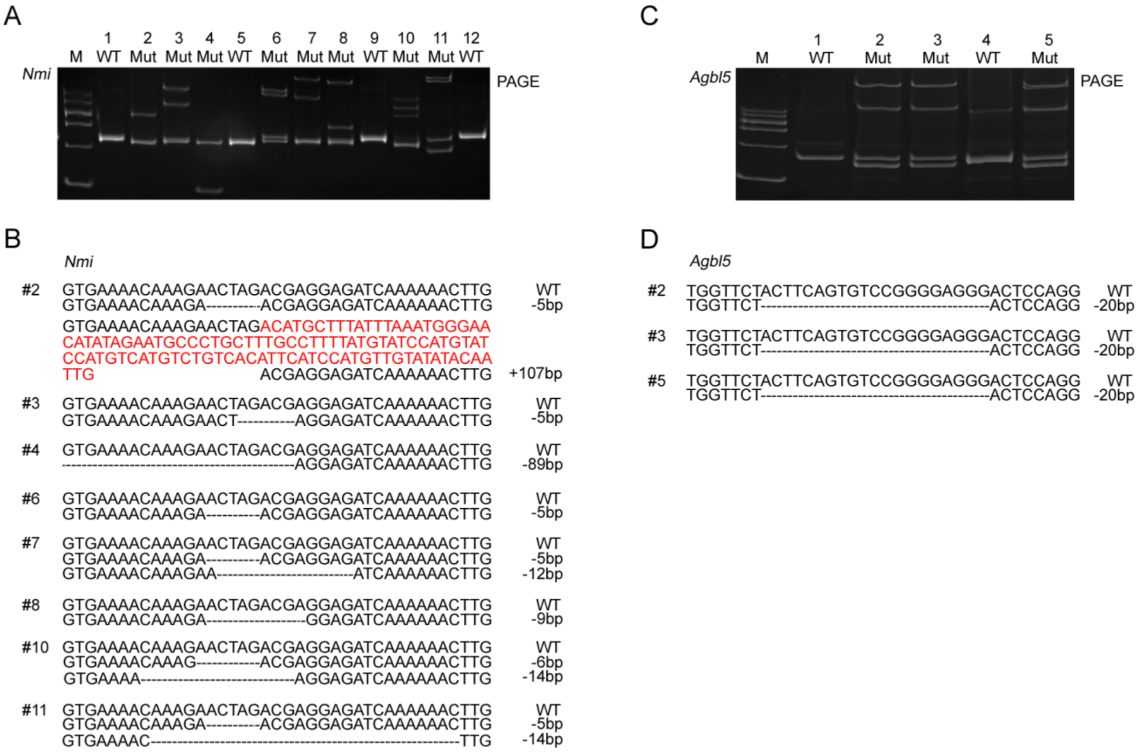

Figure S1. Detection of heteroduplex DNA in genome-modified F0 mice. (A) Screening genome-modified F0 mice in which the *Nmi* locus was targeted using the PAGE-based genotyping protocol. Eight mice displayed heteroduplex DNA pattern as observed by 15% PAGE analysis. (B) Purified PCR products from Figure S1A were further cloned for sequencing analysis. (C) Genome-modified F0 mice, in which the *Agbl5* locus was targeted, were screened using the PAGE-based genotyping protocol. Three mice displayed heteroduplex DNA patterns as observed by 15% PAGE. (D) Purified PCR products shown in Figure S1C were cloned for sequence analysis.

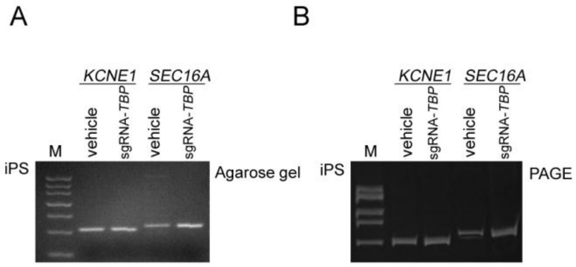

Figure S2. Off-target screening in human induced pluripotent stem cells. (A) Off-target screening using agarose gel electrophoresis. (B) Off-target screening using PAGE-based approach. Human iPSCs were electroporated with Cas9-eGFP plasmids with either vehicle (no sgRNA), or sgRNA-*TBP* (sgRNA targeting the *TBP* locus) for screening off-target loci. Two off-target loci including *KCNE1* and *SEC16A* were identified using CRISPR Design Tool (http://crispr.mit.edu).

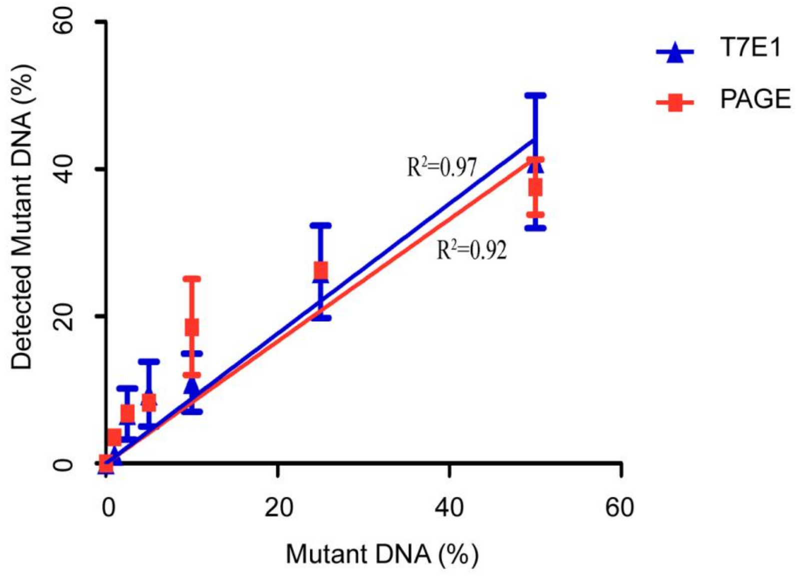

Figure S3. Quantitative analysis of PAGE-based approach and T7E1 assay. Data points measured from Figure 6 are plotted as mean ± s.e.m. of two independent experiments.
